# PD-1/PD-L1 Checkpoints and Resveratrol: A Controversial New Way for a Therapeutic Strategy

**DOI:** 10.3390/cancers13184509

**Published:** 2021-09-07

**Authors:** Dominique Delmas, François Hermetet, Virginie Aires

**Affiliations:** 1 Université de Bourgogne Franche-Comté, F-21000 Dijon, France; virginie.aires02@u-bourgogne.fr; 2 Bioactive Molecules and Health Research Group, Institut National de la Santé et de la Recherche Médicale (INSERM) Research Center U1231—Cancer and Adaptive Immune Response Team, F-21000 Dijon, France; 3 Centre Anticancéreux Georges François Leclerc Center, F-21000 Dijon, France; 4 Cancer Campus Gustave Roussy, Institut National de la Santé et de la Recherche Médicale (INSERM) UMR1287, “Hematopoietic Stem Cells and the Development of Myeloid Malignancies” Team, Université Paris-Saclay, Gustave Roussy, F-94805 Villejuif, France; francois.hermetet@gustaveroussy.fr

**Keywords:** resveratrol, polyphenols, PD-1, PD-L1, immunotherapy

## Abstract

**Simple Summary:**

Over the last decade, immunotherapies using antibodies targeting the programmed cell death 1 (PD-1) checkpoint or its ligand, programmed death ligand 1 (PD-L1), have emerged as promising therapeutic strategies against cancer. However, some current limitations include a relatively low rate of “responders”, the high cost of the treatment, and a rare risk of hyper-progression. Currently, the main challenge is, therefore, to improve these therapies, for instance, by using combined approaches. Here, we summarize the accumulating evidence that resveratrol (RSV) plays a role in the modulation of the PD-1/PD-L1 axis in cancer cells, suggesting the potential of therapeutic strategies combining RSV with PD-L1 or anti-PD-1 inhibitors. We then discuss the therapeutic potential of polyphenols such as RSV to be used in combination with PD-L1 or PD-1 inhibitors for the management of cancer patients.

**Abstract:**

Immune checkpoints refer to a range of immunoregulatory molecules that modulate the immune response. For example, proteins expressed at the surface of T-cells (including PD-1 and CTLA-4) and their ligands (PD-L1 and B7-1/B7-2, respectively), expressed by cancer cells and antigen-presenting cells, are needed to prevent excessive immune responses. However, they dampen anti-tumor immunity by limiting T-cell activity, making them promising therapeutic targets in cancer. Although immunotherapies using checkpoint blocking/neutralizing antibodies targeting PD-L1 or PD-1 have proven their superiority over conventional chemotherapies or targeted therapies by enhancing T-cell-mediated anti-tumor immunity, some limitations have emerged. These include a relatively low rate of “responders” (<50%; irrespective of cancer type), the high cost of injections, and a rare risk of hyper-progression. For clinicians, the current challenge is thus to improve the existing therapies, potentially through combinatory approaches. Polyphenols such as resveratrol (RSV), a trihydroxystilbene found in various plants and an adjuvant in numerous nutraceuticals, have been proposed as potential therapeutic targets. Beyond its well-known pleiotropic effects, RSV affects PD-L1 and PD-1 expression as well as PD-L1 subcellular localization and post-translational modifications, which we review here. We also summarize the consequences of PD-1/PD-L1 signaling, the modalities of their blockade in the context of cancer, and the current status and limitations of these immunotherapies. Finally, we discuss their potential use in combination with chemotherapies, and, using RSV as a model, we propose polyphenols as adjuvants to enhance the efficacy of anti-PD-1/anti-PD-L1 immunotherapies.

## 1. Introduction

Despite many therapeutic advances, the number of cancer cases has continued to increase in recent years. This unfortunate trend underscores failures in both prevention and treatment. In terms of disease management, therapeutic failures are often the result of the ability of cancer cells to develop various resistance mechanisms. More particularly, it appears that metabolic reprogramming has a preponderant role in the development of these mechanisms. It supports the strong replicative potential of cancer cells, allowing them to adapt to the constraints of their microenvironment. Among the new strategies that have appeared in the past 10 years to counteract chemoresistance, immunotherapy, which works through stimulation of the immune system, represents an innovative anti-cancer treatment. Indeed, the immune cells seem to recognize many types of cancer. This phenomenon, known as immunosurveillance, which was controversial at first, was clearly demonstrated in mouse tumor models developed by Robert Schreiber’s team. Tumor immunosurveillance can prevent tumor growth by killing the transformed cells before the constitution of established tumors [1]. In patients with ovarian or colon carcinoma, tumor infiltration by effector T-cells was shown to be correlated with a good prognosis and longer survival [2,3]. The concept of immunosurveillance in humans has been proven by epidemiological studies in immunodeficient patients, which revealed that these individuals had an increased cancer risk. Similar to genetically modified murine models, patients with primary immune deficiency are more susceptible to developing cancer and, more particularly, virus-induced cancers: non-Hodgkin’s lymphoma (induced by the Epstein-Barr virus, EBV), Kaposi’s sarcoma (induced by the human herpesvirus 8, HHV8) and urogenital cancers (induced by human papillomavirus, HPV) [4]. Patients with secondary immune deficiency are also likely to develop cancer faster than immunocompetent patients. It is now known that treatments targeting tumor necrosis factor alpha (TNFα) and methotrexate, which are used for their immunosuppressive effect to fight chronic inflammatory diseases (rheumatoid arthritis, inflammatory bowel diseases, systemic lupus erythematosus) [5,6] or transplant rejection, predispose the organism to oncogenesis [7]. This is also true for patients with human immunodeficiency virus (HIV), who have a low T lymphocyte count and lose the protection of T-CD4^+^ cells.

The immune system’s goal is to spot and destroy foreign bodies or cells it deems abnormal, such as cancer cells. To differentiate a cell from the “self” from a cancer cell, the immune system relies on proteins on the cell surface, called immune checkpoints. If the immune system recognizes these proteins, the immune defenses are inactivated, and the cell can continue to grow in the human body. The purpose of these checkpoints is twofold: (1) stopping the immune system’s response after its task is complete (for example, destroying a virus); (2) preventing the immune system from turning on itself and attacking normal cells, which is what occurs in autoimmune diseases. Cancer cells sometimes succeed in outsmarting the immune system by activating immune checkpoints so that they cannot be identified and destroyed. The immune system, therefore, does not respond to these cells, which then have the possibility to grow. Non-specific, adoptive, and active immunotherapy strategies show moderate efficacy, which is explained by the common point that characterizes them all: their sole objective is to massively stimulate immunity against the tumor. Nonetheless, but fortunately for us, our immune system is equipped with a set of self-inhibiting mechanisms that prevent the dire consequences of a chronic and uncontrolled inflammatory response. This self-inhibition, which follows the activation of the immune system, involves a large number of membrane receptors that act as an immunological checkpoint, more commonly called an “immune checkpoint”. The recent discovery of these checkpoints was made possible thanks to the study of the molecular mechanisms involved in the primary or secondary activation of B, natural killer (NK), and T lymphocytes. Among these checkpoints is that of the PD-1/PD-L1 combination. Anti-PD-1 and anti-PD-L1 immunotherapies or checkpoint inhibitors act on the junction between the immune cell (T lymphocyte) and the proteins that have developed on the surface of the cancer cells.

## 2. Overview of PD-1/PD-L1 System

The programmed cell death-1 (PD-1)/programmed death-ligand 1 (PD-L1) pathway regulates T-cell activation and function and controls the induction and maintenance of cell-based immune tolerance within the tumor microenvironment. The activation of PD-1 on T-cells by its ligands, PD-L1 or PD-L2, are responsible for impairing T-cell activation, proliferation, and cytotoxic secretion, leading to suppression of effective anti-tumor immune responses in cancer.

PD-1 (CD279) is an inhibitory receptor discovered in 1992 by a team that was initially looking for new genes involved in programmed cell death in two hematopoietic cell lines (LyD9 and 2B4.11) [8]. They found that activation by phorbol-myristate-acetate (PMA) and ionomycin caused the death of these cells, and they revealed that the PD-1 gene was upregulated in a cDNA library analysis done just before the death process. However, the direct role of PD-1 in the process of programmed cell death was finally invalidated, and its role remained unrecognized until 1998. Honjo’s work described the spontaneous onset of autoimmune diseases such as lupus in PD-1-deficient mice (Pdcd1^−/−^) [9]. PD-1 was identified on the surface of T-CD4^+^, T-CD8^+^, B, or NK lymphocytes during their activation and on certain myeloid cells such as dendritic cells and monocytes. PD-1 expression is induced through the three activation signals of the lymphocyte. B-cell or T-cell receptors (respectively, BCR and TCR) promote its expression through the Zeta-chain-associated protein kinase 70 (ZAP70)/phospholipase C gamma (PLCγ) pathway and the transcription factors activating protein 1 (AP1), nuclear factor of activated T-cells (*NFAT*), and nuclear factor-kappa B (NF-κB), which induce PD-1 gene expression. The signal induced by CD28 causes the activation of the phosphoinositide 3-kinase (PI3K)/protein kinase B (Akt) pathway, which helps support TCR-induced pathways. Finally, PD-1 expression can also be stimulated by cytokines such as interferon gamma (IFN-γ) or by interleukins such as IL-2, IL-7, IL-15, and IL-21 through the activity of the transcription factors signal transducer and activator of transcription 1 and 2 (STAT1/STAT2) as well as through the transcription factors interferon regulatory factor 1 and 9 (IRF1/IRF9) [10]. The pathways triggered by PD-1 within the T lymphocyte are induced by its cytoplasmic tail, composed of an immunoreceptor tyrosine-based inhibitory motif (ITIM) and an immunoreceptor tyrosine-based switch motif (ITSM) [11]. These motifs make it possible to inhibit the immune response via phosphatases, such as SHP-2, which inhibit the lymphocyte-specific protein tyrosine kinase (LCK)/ZAP70/PI3K pathway, leading to a sharp decrease in proliferation (drop in Ki67 expression), cytotoxic activity (Granzyme, Perforin), and the production of Th1 type cytokines (IFN-γ, TNFα, IL-2) [12].

The ligands for PD-1 are PD-L1 (B7-H1, CD274) and PD-L2 (B7-DC or CD273). PD-L1 can be expressed by activated T lymphocytes and myeloid cells but also by normal or cancerous endothelial and epithelial cells. PD-L2 is expressed more restrictively than PD-L1 and is mainly found on the surface of dendritic and tumor cells [13,14,15]. The presence of PD-L1 on the surface of non-hematopoietic cells indicates that the role of PD-1 may be in peripheral tissues and that, therefore, it may play an important role in tumor immunity. Expression of PD-L1 by tumor stromal cells or by the cancer cells themselves may promote the escape of the tumor from the immune system. Histological studies of many types of cancer show that a high expression of PD-L1 is associated with more advanced cancers with a poorer prognosis [16,17]. Two mechanisms of resistance to immunity involving the PD-1/PD-L1 pathway exist and can co-exist: innate resistance and adaptive resistance. Tumor cells can constitutively express inhibitory receptor ligands on their surface following genetic deregulation or in response to inflammatory signals present in their environment (IFN-γ). The constitutive expression of PD-L1 has been found in many types of cancer (breast, glioma, prostate, lung). This phenomenon is linked to the constitutive activation of various oncogenic pathways such as the PI3K/AKT, ALK, STAT3, and even mitogen-activated protein kinase (MAPK) pathways [18,19]. The development of adaptive resistance involves physiological PD-L1 induction mechanisms that arise during stimulation by IFN-γ to protect tissues from an excessive immune response. It is in the latter case that stromal cells may also participate in the inhibition of the cytotoxic response. The signaling pathways that make it possible to induce PD-L1 by IFNγ involve the activation of the Janus kinase 2 (JAK2)/STAT1, P13K/AKT, and Ras/MAPK pathways, which induce the gene expression of PD-L1 via the IRF1, cJun, cFos, and NF-κB transcription factors [20]. IFN-γ is not the only cytokine to induce PD-L1. Type I and II interferons, TNFα, ILs-2, 7, 10, 12, 15, and 21 can also induce its expression.

## 3. Disruption of the PD-1/PD-L1 Pathway with Specific Antibodies

The PD-1/PD-L1 bond, therefore, occupies a central role in the efficiency of the immune system. Scientists have thus focused their efforts on finding a way to restore the action of the immune system against tumor cells despite this binding.

To prevent PD-1 and PD-L1 proteins from binding together, researchers have developed antibodies capable of binding to PD-1 or PD-L1, called anti-PD-1 or anti-PD-L-1 antibodies (also called anti-PD-1 or anti-PD-L1 immunotherapies). Blocking the immune checkpoint by preventing the interaction between PD-1/PD-L1 prevents the inactivation of T-cells and restores key T-cell effector functions, which are then able to fight the tumor cells. The availability of these new treatments is relatively recent. They were first used on certain types of skin and lung cancers.

### 3.1. Anti-PD-1 Antibodies

Anti-PD-1 antibodies, nivolumab (Opdivo©) or pembrolizumab (Keytruda©), block the binding of PD-1 with its two lineages, PD-L1 (B7-H1, CD274) and PD-L2. Nivolumab has a proven objective response in several types of cancer (melanoma, kidney, and lung cancer), and it was the first anti-PD-1 antibody to obtain marketing authorization in 2014 in Japan for advanced unresectable melanoma. Anti-PD-1, and nivolumab, in particular, have shown very significant efficacy in melanoma [21]. A study of 418 patients with advanced melanoma showed that nivolumab was superior to dacarbazine. In the nivolumab group, the complete response rate was 8%, while it was 1% in the dacarbazine group. The objective response rate, as well as the duration of response, was also much higher in the nivolumab group (32% versus 13% for objective response; median duration of response not reached versus 6 months) [22]. In non-small cell lung cancer (NSCLC), the CheckMate 057 study, which compared nivolumab to docetaxel, showed an increase in median overall survival. This trial also showed an objective response rate of 19% for nivolumab versus 12% for docetaxel. Similarly, the CheckMate 017 study compared nivolumab with docetaxel in advanced bronchial squamous cell carcinomas that progressed after a first line of chemotherapy with platinum salts. This study confirmed that nivolumab had better results in terms of overall survival. The objective response rate was 20% for nivolumab compared to 9% for docetaxel. Finally, progression-free survival was in favor of nivolumab (HR = 0.62 (95% CI, 0.47–0.81; *p* < 0.001)) [23]. Nivolumab thus obtained its marketing authorization for the treatment of metastatic melanoma in 2014 and for the treatment of NSCLC and kidney cancer in 2015. Since then, another anti-PD-1 antibody, pembrolizumab, has received marketing authorization. In 2016, the FDA also cleared the use of atezolizumab (anti-PD-1) for the treatment of bladder cancer and the use of nivolumab for Hodgkin’s lymphoma. Current strategies also aim to combine antibodies that neutralize inhibitory receptors. In 2015, the FDA announced that it would accelerate the acceptance of an anti-CTLA-4/anti-PD-1 combination for the treatment of metastatic melanoma, following results published in June 2015 indicating that this combination made it possible to induce a stronger response than with the two monotherapies [24].

### 3.2. Anti-PD-L1 Antibodies

Anti-PD-L1 antibodies have also shown significant therapeutic efficacy in the treatment of various cancers. Phase I of MEDI 4736 showed objective responses in melanoma (17%), lung (10%), ovarian (6%), and kidney (12%) cancer [23]. Other studies have shown clinical benefit in kidney cancer, lung cancer, and melanoma [25,26,27]. Among them, atezolizumab (Tecentriq^©^) is used for urothelial carcinoma, NSCLC, and triple-negative breast cancer, and avelumab (Bavencio^©^) is indicated for Merkel cell carcinoma.

### 3.3. Limitations of Immunotherapies and Combination with Chemotherapy

Today, there are many indications for immunotherapy, and many patients can benefit from them. Scientists are currently conducting further studies to assess the efficacy and tolerance of these molecules in other types of cancer, alone or in combination with other treatments. Immunotherapies are most often prescribed if previous lines of treatment have failed (chemotherapy, for example), and they are also sometimes approved in the first line, alone or in combination. Unfortunately, the use of antibodies directed against inhibitory receptors does not yet induce prolonged responses in the majority of patients. However, preclinical studies show that it is possible to amplify the therapeutic response by combining the blocking of immunomodulatory receptors with more “conventional” therapies. There are many unanswered questions regarding the optimal administration schedule for immunotherapy and combination therapy. In order to increase clinical response rates, it is important to address how and when to use combination therapies. Oncologists now have a diverse armamentarium (active immunotherapy, targeted therapy, radiotherapy, chemotherapy), and combining these therapies with inhibitory receptor blockers could be a successful strategy. The therapeutic effect of chemotherapy works not only through direct cytotoxicity to tumor cells but, in certain cases, through the activation of immunity. Indeed, chemotherapies can promote antigenicity (expression of CHM-I, activating or inhibiting ligands of NKs) and immunogenicity (immunogenic cell death).

A recent study has shown that immunogenic chemotherapy (oxaliplatin) associated with inhibitory chemotherapy of Treg (cyclophosphamide) can sensitize tumors originally resistant to immunotherapy (anti-PD-1 and anti-CTLA-4). Conversely, the use of non-immunogenic chemotherapy (cis-platinum and paclitaxel) does not provide a synergistic effect. Histological tumor analysis (spontaneous lung tumor, K-Ras/Trp53) has shown that T lymphocytes are present in the periphery of the tumor, close to the blood vessels. Treatment with immunogenic chemotherapy allows these cells to be recruited into the heart of the tumor. This mechanism is dependent on immunogenic tumor cell death, which promotes the recruitment and activation of dendritic cells through toll-like receptor 4 (TLR4). The chemotherapy used also modulates the T-CD8^+^/Treg ratio, and the inhibition of Treg by cyclophosphamide certainly enhances the effect of immunotherapy by limiting immunosuppression [28]. Similar results have been obtained with other chemotherapy drugs and in other cancer models. In a mesothelioma model, the combination of gemcitabine + anti-CTLA4 induced a massive infiltration of T-CD4^+^ and T-CD8^+^ lymphocytes expressing the inducible T-cell COStimulator (ICOS) activation marker and the Ki67 proliferation marker. The therapeutic effect of this combination is powerful, resulting in 50% complete regression [29]. The molecular mechanisms behind the effects of this combination are not described in this study, but we can hypothesize that the use of gemcitabine limits the expansion of immunosuppressive myeloid cells, thereby eliminating an additional immunosuppressive mechanism with the blockade of CTLA-4. Identical results were obtained with the combination of surgery/gemcitabine/anti-CD40 (anti-CD40 is an activating antibody) [30]. Unpublished results obtained in our laboratory show that the anti-PD-1/5-FU/oxaliplatin combination achieves approximately 40% tumor regression in MC38 and CT26 colon cancer models. As with the oxaliplatin/cyclophosphamide combination, this combination induces the recruitment of cytotoxic CD8^+^ producing IFN-γ and expressing the activation markers CD69, CTLA4, PD-1, and Tim-3. The recruitment of these cells also involves the induction of immunogenic death, which in turn, promotes a specific tumor antigen response (AH-1 for the CT26 line and SIINFEKL for the MC-38-OVA and CT26-OVA line). It is also important to note that the use of 5-FU decreases the presence of immunosuppressive myeloid cells in the tumor and spleen. However, despite significant intratumoral T-CD8^+^ infiltration, the therapeutic efficacy of the 5-FU/oxaliplatin combination remains transitory. This effect is, in part, due to the adaptive immunity resistance mechanism. By infiltrating the tumor, T-CD8^+^ cells will reduce tumor growth by secreting proteases and cytotoxic cytokines (IFN-γ). The production of IFN-γ will slow down tumor growth, but it will also promote the expression of PD-1 and PD-L1. These two molecules can then stop the immune reaction and allow the tumor to escape immunosurveillance once again. This biological process opens the door to a new sequential therapeutic combination: 5-FU/oxaliplatin, then anti-PD-1 or anti-PD-L1. In the CT26 and MC38 models, this strategy results in significant regression rates, while chemotherapy or immunotherapy used alone cannot achieve the same rates [31]. These preclinical studies are still few in number, and a lot of work is needed to determine what may be the best combinations and the best administration schedules.

Some clinical trials are underway to accurately assess the effectiveness of these combinations. Recent studies in humans have shown that these treatment strategies are promising. In 2011, Caroline Robert’s team showed that the dacarbazine/ipilimumab combination has a greater effect compared to placebo/ipilimumab. The overall survival at 1 year was 47.3% versus 36.3%, and at 2 years, it was 28.5% versus 17.9% [32]. The use of dacarbazine in combination with an antibody targeting inhibitory receptors present on the surface of NK cells could also be of interest. In addition, our team (Ghiringhelli et al.) showed that this chemotherapy induces the expression of NKG2D ligands (MICA, MICB, ULBP in humans and RAE1, H60, and MULT1 in mice), which sensitizes tumor cells to NK-induced lysis. The combination of this effect and an inhibitory receptor blockade (KIR) could have an increased therapeutic impact [33]. In lung cancer, the activity of nivolumab is not the same when combined with gemcitabine/cisplatin, pemetrexed/cisplatin, or paclitaxel/carboplatin. The 2-year survival rates indicate that the best combination is paclitaxel/carboplatin, with 62% survival versus 25% and 33% in the gemcitabine/cisplatin and pemetrexed/cisplatin groups [34]. This study was not intended to explain why the paclitaxel/carboplatin combination was more effective, but one would think that the immunogenic effect of this combination is superior to the others.

In general, anti-PD-1 or anti-PD-L1 immunotherapies are better tolerated than chemotherapy: they do not cause hair loss and cause less nausea and fatigue. However, rare (less than 1% of cases) but potentially serious side effects have been reported, mainly neurological (neuropathies, neuromuscular disorders, encephalopathies) or hematological (neutropenia, anemia, thrombocytopenia) complications. The adverse effects induced by anti-PD-1/PD-L1 antibodies are more varied, less predictable, and less classic. These complications are immune-related—that is to say that they are caused by an imbalanced immune system that begins to attack its own cells.

## 4. Polyphenols as Adjuvants to Enhance Anti-PD-1/Anti-PD-L1 Immunotherapies

The immune response in situ is an important factor in the response to immunotherapy. In the context of treatments targeting the PD-1/PD-L1 pathway, the analysis of PD-L1 expression in tumors (melanoma, lungs) appears to be relevant since it could be used to identify the patients who would most benefit. The expression of PD-L1 on the surface of immune cells present at the margin of the invasion is also strongly correlated with a better response, particularly if this expression is associated with significant infiltration of CD8^+^ cells [35,36]. In their review, Liu et al. summarize the biophysical and biochemical assays employed for the measurements of the binding capacities, molecular interactions, and blocking effects of small molecule inhibitors on the PD-1/PD-L1 system [37]. Among these small molecules, polyphenols could be good candidates. Indeed, because of their ability to influence both the immune system and the molecular mechanisms of tumor and immune cells, polyphenols could act as modulators of the PD-1/PD-L1 system, as previously mentioned by Hsieh and M. Wu [38], and, thus, be good candidates for a combination with anti-PD-1/anti-PD-L1 with or without chemotherapy [39,40,41].

**Figure 1:**
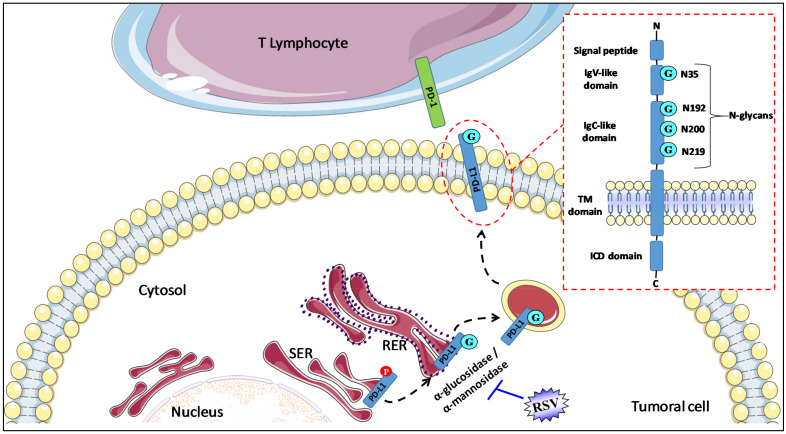
RSV alters N-glycosylation to disrupt PD-1/PD-L1 interaction. PD-L1 is composed of a transmembrane region (TM domain) and two extracellular domains, the IgC-like and IgV-like domains. The short intracytoplasmic domain (ICD domain) of PD-L1 is responsible for the triggering of the signaling pathways inside cells. N-glycosylation is a major post-translational modification of PD-L1 that is crucial for its stability (prevention of PD-L1 degradation through GSK-3β-mediated 26S proteasome), intracellular trafficking, and functions (protein–protein interactions). Four glycosylation sites of asparagine residues (G) span within the IgV-like (1 site, N35) and IgC-like domains (3 sites of glycosylation, N192, N200, and N219). There is a ≈17 kDa shift in PD-L1 molecular weight between its non- (33 kDa) and N-glycosylated form (50 kDa). RSV interacts with and blocks α-glucosidase/α-mannosidase in tumoral cells and prevents glyco-PD-L1-processing, which, in turn, promotes the retention of the abnormally glycosylated form of PD-L1 in the endoplasmic reticulum. Ig-V, immunoglobulin variable; IgC, immunoglobulin constant; SER, smooth endoplasmic reticulum; RER, rough endoplasmic reticulum; TM, transmembrane; ICD, intracellular domain.

Polyphenols constitute a wide range of plant-derived compounds present in the human diet that may protect against vascular diseases, cancers, and associated inflammatory effects [42,43,44,45]. For instance, several cohort studies have demonstrated a significant inverse association between flavonoid consumption and cardiovascular risk [46]. Other epidemiological studies suggest that phytoconstituents or micronutrients have a protective effect against cancer [47]. Levi et al. showed, for instance, an inverse relation between resveratrol (RSV; a non-flavonoid polyphenol) and breast cancer risk [48]. Moreover, a link between flavonol consumption and reduced risk of lung cancer was also demonstrated in Finland with a cohort study [49]. These reports have reinforced the idea that polyphenols and flavonoids have beneficial health effects. These compounds exhibit variable antioxidant, anti-inflammatory, and anti-cancer properties that depend on their structure, which determines their stability, permeability, and affinity with their target (i.e., plasma membrane, enzymes, or DNA). Moreover, some polyphenols can modulate the expression of PD-1/PD-L1 or can alter its pathway. This is especially true for RSV, which is a trihydroxystilbene found in various plants, used as an adjuvant in numerous nutraceuticals. This polyphenol has pleiotropic effects, and it could affect various signaling pathways involved in the control of PD-L1 and PD-1 expression. Indeed, it is often suggested that RSV affects the PD-1/PD-L1 system through the modulation of the kinase activation pathways, but few articles have shown both the expression of PD-L1/PD-1 and the associated modulation of these targets. In this review, we focus only on data that has demonstrated an association between the modulation of PD-1/PD-L1 expression and the molecular pathways affected by RSV. For this analysis, a systematic search of PubMed (https://pubmed.ncbi.nlm.nih.gov/ accessed on 30 June 2021) was conducted to identify studies conducted with RSV or its metabolites in the PD-1/PD-L1 pathway, on experimental cells and animals, or in humans in relation to immunity/oncology, up to August 2021. The search term “resveratrol” was used in combination with “PD-1” and “PD-L1”. Only 10 original articles were found with the combination of “resveratrol” and “PD-L1” and only 6 original articles with “resveratrol” and “PD-1”. These studies were conducted in various domains such as oncology, pharmacology, metabolism, or nutrition, and some looked at the potential usefulness of this treatment in several disorders (Figure 1). In this review, we focus only on original articles that clearly demonstrate a correlation between a modulation of the expression of PD-L1 or PD-1 and a treatment with RSV or its metabolites, thus setting aside the articles for which extrapolations are made based only on the known properties of RSV but not correlated with a modulation of PD-1 or PD-L1. A complementary approach was used to analyze RSV bioavailability and clinical trials using RSV. The search was limited to English-language sources.

### 4.1. Resveratrol (RSV) and PD-1/PD-L1 Expression

In lung cancer, PRI-219, a vitamin D3 active metabolite (24R-1,24-dihdroxycholecalciferol), in combination with a hydroxystilbene, RSV (3,5,4′-trihydroxystilbene), induced a significant increase in PD-L1 mRNA expression in NCIH358, A549, and HCC827 cells when compared to PRI-2191 treatment alone [50]. RSV alone also had a non-significant tendency to upregulate PD-L1 expression in HCC827 cells [50]. Similarly, RSV was able to increase PD-L1 expression in two lung adenocarcinoma cell lines (A549 and H1299) [51]. An increase in PD-L1 expression was also observed with RSV and one of its metabolites, piceatannol (3′,4′,3,5-tetrahydroxystilbene), in human breast cancer (BT549, SKBR3), invasive ductal carcinoma (BT474), and colorectal cancer (HT29, SW480, SW60, HCT116) cells [52]. Very interestingly, the treatment of cells with the two stilbenoids induced a synergistic upregulation of PD-L1. Moreover, Lucas et al. showed that a low endogenous mRNA level of PD-L1 is more likely to be affected by RSV and piceatannol alone or in combination [52]. The differential increase in PD-L1 expression induced by RSV or piceatannol was observed in 2/4 breast and 3/4 colorectal cancer cell lines treated with either of the stilbenoids alone. In addition, in non-small cell lung cancer (NSCLC) cell lines, the increase in PD-L1 expression was associated with a suppression of T-cell function in a coculture model [51]. In fact, RSV upregulated PD-L1 by activating the WNT pathway, which is consistent with reports showing that a dysfunctional WNT pathway alters PD-L1 expression in triple-negative breast cancer [51,53]. RSV was shown to activate sirtuin-1 (Sirt-1), which induces the deacetylation and stabilization of the transcription factor Snail, which, in turn, inhibits the transcription of Axin 2. Subsequently, the authors observed the disassembly of the destruction complex and the enhanced binding of β-catenin/TCF to the PD-L1 promoter [51].

Conversely, RSV was shown to antagonize thyroid-hormone-induced PD-L1 expression in oral cancer cells at relatively high concentrations [54] and to significantly reduce PD-L1 expression in oral epidermoid cancer cells [55]. Very interestingly, these effects were associated with an accumulation of nuclear PD-L1.

These controversial data must be balanced with the results of Verdura et al., showing that the localization of PD-L1 is crucial for its functioning [56]. Therefore, in their model, the authors attempted to decipher whether the enhanced up-regulation of PD-L1 in lung cancer cells was associated with PD-L1 restraint in cytoplasmic compartments or on the plasma membrane.

### 4.2. RSV and Key Regulators of PD-1/PD-L1 Pathways

#### 4.2.1. RSV and Glycosylation of PD-L1

Protein structure and function are heavily determined by *N*-glycosylation, and glycosylated PD-L1 is found in various cancer models [57]. *N*-linked glycosylation is a sequential reaction that begins in the endoplasmic reticulum (ER), in which the oligosaccharyltransferase (OST) complex transfers a 14-sugar moiety, Glc_3_Man_9_GlcNAc_2_ (Glc, glucose; Man, mannose; GlcNAc, *N*-acetylglucosamine), from dolichol lipid to the Asn residue in the consensus Asn-X-Ser/Thr motif within the nascent polypeptide chains (X denotes any amino acid except proline) [58,59]. Glycosylation prevents the degradation of PD-L1 by the proteasome and increases the stability of the protein, but its regulation is altered by oncogenic processes. Indeed, PD-L1 phosphorylation is associated with its glycosylation [60]. N-glycosylation of PD-L1 affects its interaction with PD-1. Using molecular modeling of the PD-L1/PD-1 interaction with N-glycans, Benicky et al. have shown that glycans at the N219 site of PD-L1 and N74 and N116 sites of PD-1 may be involved in glycan–glycan interactions [61]. In view of the importance of glycosylation in the functionality of PD-L1, strategies aimed at modulating this function have been deployed, in particular, with monoclonal antibodies, directed specifically against the glycosylated forms of PD-L1, whose anti-tumor action seems promising in preclinical models [62].

Menendez’s team has shown that RSV could act as a direct inhibitor of the glyco-PD-L1-processing enzymes (α-glucosidase/α-mannosidase) that modulate the *N*-linked glycan decoration of PD-L1, thereby promoting the retention of a mannose-rich, abnormally glycosylated form of PD-L1 in the endoplasmic reticulum [56]. Interestingly, the use of GSK3 beta inhibitors (AR-18, LiCL) failed to reverse the RSV-induced migration pattern of PD-L1, thereby ruling out the possibility that RSV might indirectly disrupt N-linked glycosylation through GSK3β activation [56]. Moreover, this disruption of PD-L1 N-glycosylation was also independent of sirtuin-1 (SIRT-1) and AMPK. These events led to a decrease in cell-membrane-associated PD-L1 and the apparent retention of PD-L1 in perinuclear compartments. Using computer-aided docking/MD simulations, the authors predicted that RSV would bind to the PD-L1 dimer surface, subsequently showing an increase in the susceptibility of cancer cells to T-cell-mediated cell death [56].

#### 4.2.2. RSV and NF-kB

**Figure 2:**
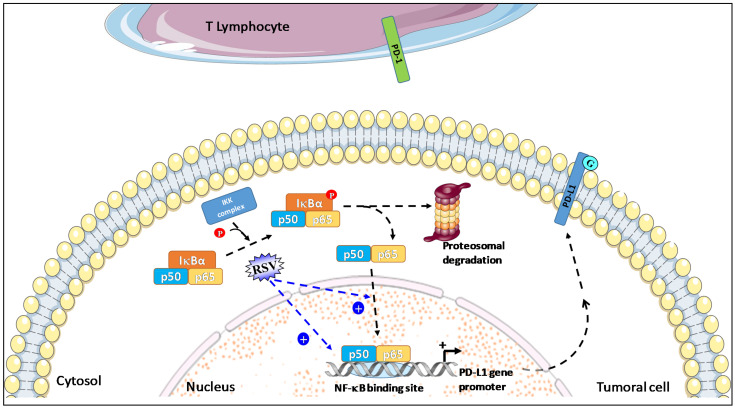
RSV used in combination with piceatannol leads to a synergistic induction of NF-kB expression and increases the translocation and nuclear accumulation of NF-kB p50 and p65 subunit in tumor cells. Activated NF-kB pathways then induce PD-L1 gene transcription by the binding of the p50 and p65 subunits to its promoter and can also regulate PD-L1 post-transcriptionally.

NF-kB has been described as a key positive regulator of PD-L1 expression in cancer because it directly induces PD-L1 gene transcription by binding to its promoter; NF-kB is also able to regulate PD-L1 post-transcriptionally through indirect pathways [63]. Following the activation of the canonical pathway, including IkB phosphorylation through the IkB kinase (IKK), IkB is ubiquitinated and targeted to degradation by the proteasome. Finally, NF-kB is translocated into the nucleus, from which it regulates PD-L1 gene transcription by binding to its promoter (Figure 2).

In the study by Lucas et al. using an A549 NF-kB reporter assay, a synergistic induction of NF-kB expression was obtained by piceatannol alone and in combination with RSV (Figure 2). Moreover, these stilbenoids increase the translocation and nuclear accumulation of p65 [52]. The induction of PD-L1 expression at the surface of colorectal cancer SW620 cells by RSV or a mixture with stilbenoids was significantly decreased when cells were co-treated with an IKK kinase inhibitor such as BMS-345541 [52,64], suggesting that NF-kB activation is involved in the induction of PD-L1 expression by these stilbenoids. Based mainly on this observation, Hsieh and M. Wu proposed that RSV/piceatannol-mediated PD-L1 upregulation may act as a “Search, Enhance, and Engage” signal for an anti-PD-1/PD-L1 immune checkpoint blockade, suggesting that beneficial response rate to this immunotherapy may be enhanced when combined with certain dietary polyphenols such as RSV and piceatannol [38].

These results are surprising because in other cell types, in particular in cancer cells, RSV is described as an inhibitor of the NF-kB pathway. Indeed, this hydroxystilbene was able to reduce the nuclear content of NFκB subunits and the nuclear translocation of the p65 subunit of NFκB, and it suppressed the phosphorylation and degradation of IκBα, resulting in its retention in the cytoplasm [65,66,67,68]. Thus, by disrupting this nuclear factor and the NF-kB pathway, RSV affects the expression of various genes involved in tumor growth. These genes include *inos* (inducible nitric oxide synthase) or *cox-2* (cyclo-oxygenase synthase-2), which are partly controlled by NFκB [69]. These divergent results are likely cell-type dependent, but more studies are warranted to determine whether RSV affects immune cell genes and cancer cell genes in the same way.

#### 4.2.3. RSV and Histone Deacetylase

The expression of PD-L1 can be regulated via histone acetylation/deacetylation [70,71]. Histone deacetylases (HDACs) are key mediators of epigenetic regulation. Their role is to remove acetyl groups from the N-acetyl lysine amino acid on the tail of histones, and they appear promising as therapeutic targets for cancer. It should be noted that both alone and in combination with conventional chemotherapy, HDAC inhibition increases tumor cell PD-L1 expression [72].

Therefore, the use of HDAC inhibitors (i.e., vorinostat, mocetinostat, resminostat, and entinostat) or inhibitors of histone acetyltransferase (HAT) (i.e., curcumin, garcinol, anacardic acid, MB-3, and Tip60i) reduced the ability of RSV and piceatannol alone or in combination to induce PD-L1 expression by metastatic colorectal SW620 cells, suggesting that the upregulation of PD-L1 by stilbenoids involves transcriptional control through the induction of HDAC [52]. These data are strengthened by the literature, which shows that RSV is an activator of HDAC in many cell types [73].

#### 4.2.4. RSV and β-Catenin/Wnt Pathway

β-catenin plays an essential role in regulating PD-L1 expression. It has been shown that depleting β-catenin reduces the expression of PD-L1 while overexpressing a constitutively active β-catenin mutant enhances PD-L1 expression [74].

By using a Wnt inhibitor (FH535), RSV-induced PD-L1 expression was significantly reduced in lung cancer cells [51]. Moreover, when the β-catenin destruction complex is destabilized, β-catenin is stabilized in the cytoplasm. It is then translocated into the nucleus, where it binds to the TCF/LEF complex to activate gene transcription. In a study by Yang et al., CHIP assays were used to reveal that the responsiveness/activity of PD-L1 promoter to RSV was reduced when the third TCF-4 binding site was mutated. Additional work showed that RSV promotes a Snail-dependent reduction of Axin2 levels, and RSV-activated SirT1 promotes Snail protein stability as a result of deacetylation [51].

### 4.3. New Developments

Chen et al. have developed dual-responsive mPEG-PLA-Phis-ss-PEI polyplexes (DRP/RSV/siP) that can be used for the robust co-delivery of PD-L1 siRNA and RSV. Very interestingly, DRP/RSV/siP downregulated glycolysis and upregulated mitochondrial oxidative phosphorylation (OXPHOS) in mouse melanoma cells and colorectal tumor cells, associated with a reduction of lactate production and glucose consumption [75]. This alteration of mitochondrial OXPHOS, which was also observed in vivo, promoted CD8^+^ and CD4^+^ T-cell infiltration and IFN-γ secretion. It also suppressed Treg cells and MDSCs at the same level (glycolysis), resulting in an enhanced anti-cancer effect when combined with PD-L1 silencing [75]. This point is fundamental since it has been demonstrated that balancing glucose metabolic pathways generates a more potent response to PD-L1 silencing than exclusively inhibiting glycolysis by shaping less immune-suppressive tumor micro-environments. Moreover, the combined use of RSV and piceatannol, co-administered with anti-PD-L1 immunotherapy, may exhibit clinical benefits in cancer patients with no- or- low-PD-L1 tumors. In ovarian carcinoma, for example, RSV stimulates immunogenic cell death, and, in vivo, it markedly inhibited tumor growth when combined with anti-PD-1 monoclonal antibody [76].

## 5. Bioavailability of Resveratrol (RSV) and Clinical Trials in Anti-Cancer Strategy

### 5.1. Biotransformation and Pharmacokinetics of RSV

There is a lack of coherence in the current literature discussing the pharmacokinetics of RSV. The abundance of disparate studies suggests that the effects are highly dependent on the target tissue and the desired effect [77].

The absorption of polyphenols is generally poor. Nevertheless, when compared to quercetin and catechin, RSV is relatively well absorbed by the intestine after oral administration [78,79]. Our team has previously studied the transport mechanisms of RSV in human hepatocytes and a hepatoblastoma cell line HepG2 [80]. Fluorescence microscopy was used to establish that polyphenols penetrate rapidly into liver cells and are distributed throughout the cell, except for the nucleus, similar to what we observed for colonic tumor cells. Using tritium-radiolabeled RSV, we also found that the polyphenol penetrates into cancer cells and hepatocytes in a time-, concentration-, and temperature-dependent manner without causing toxicity in the hepatocytes. Cis-inhibition experiments using unlabeled RSV in excess of the tracer suggest that RSV is transported into liver tumor cells by both a passive diffusion process and a facilitated transport mechanism. It is most likely, because of similarity in the uptake of radiolabeled RSV between colonic and hepatic tumor cells, that RSV is endocytosed via a raft-dependent pathway [81,82]. Fluorimetric and exclusion chromatography studies show a strong interaction between albumin and the polyphenol, with a complex formation favored by the presence of fatty acids [83]. These results are interesting from a physiological point of view, seeing as, in the general circulation, molecules can bind to plasma proteins present in large quantities to form complexes. This binding is usually reversible and in equilibrium: free molecule + proteins → molecule–protein complex. 

The binding of molecules to plasma proteins has an important physiological significance because the concentration of the free form conditions the importance of the effect and the speed of elimination. We can then hypothesize that the complex formed by albumin with trihydroxystilbene could constitute, in vivo, a plasma reserve, allowing a prolonged release of the molecule towards its cellular targets. Recent studies on the delivery of chemotherapeutic agents are currently evaluating the potential of albumin-bound in vivo anti-cancer treatments [84]. In particular, albumin binding of taxol has been shown to enhance its ability to target tumor cells and its efficacy [85]. This could be the case for RSV as well.

We were able to show that RSV is highly conjugated after 4 h of incubation in liver cells, and an analysis by HPLC and mass spectrometry revealed the presence of mono- and disulfate forms. Two isomeric forms are possible for both RSV monosulfate (RSV-3-sulfate and RSV-4’-sulfate) and RSV disulfate (RSV-3,4’-disulfate and RSV-3,5-disulfate) [86]. These different metabolites were found in the plasma of Wistar rats after oral administration of RSV; an additional metabolite, RSV-trisulfate, was also detected [87,88]. Interestingly, studies have shown that RSV is able to induce its own metabolism [86]. Indeed, a 48 h pre-treatment of liver cells with RSV (10 µM) resulted in a higher level of conjugates when RSV was added again for 2 or 4 h on these pretreated cells. This elevation could be the result of the induction of phase II RSV metabolizing enzymes. A study of the expression of UGTs (uridine diphosphate glucuronosyltransferases) and ST (sulfotransferase) showed that RSV can increase the levels of gene and protein expression of UGT 1A1 and 2B7 and of ST1E1. It appears that the conjugation of phenolic groups strongly affects cytotoxicity, and these conjugates are much less effective on breast cancer lines compared to RSV. Conversely, according to Baur and Sinclair, RSV metabolites notably retain the ability to activate SIRT1 and inhibit COX in vitro [77]. In fact, the in vitro activity of these metabolites does not necessarily reflect their in vivo activity, as there are ubiquitous sulfatases in humans that could convert the metabolites into RSV. The small number of studies focused on these molecules is likely due to their instability and, thus, their limited commercial availability. As for the transport of these molecules in the body, some studies suggest the involvement of MRP2 and MRP3, but this remains to be demonstrated [89,90].

The identification and study of the pharmacokinetics of RSV metabolites in animal models and humans (Table 1) show that RSV is metabolized into two 3 and 4’-monoglucuronide isomers, RSV-3-monosulfate, monoglucuronide and monosulfate dihydroRSV, a disulfate metabolite, and a glucuronide-sulfate [91,92].

Studies conducted in humans to investigate the pharmacokinetics of RSV, either from the pure compound or from wine or other beverages [78,79,91,92,95,99,100,101,102], have shown that RSV is rapidly absorbed after oral ingestion, with levels detectable in both plasma and urine (Table 2). In humans, the concentration of free plasma RSV reaches a maximum of 2.4 µM 1.5 h after ingestion of five grams of pure RSV and then decreases rapidly over the following 5 hours. The low circulating levels of RSV can be partly explained by the fact that RSV is rapidly metabolized in the digestive tract by intestinal and hepatic phase II enzymes, generating glucuronide and sulfate conjugates [103].

These metabolites have the same plasma half-life as RSV but have three- to eight-fold higher plasma levels for 4 h before the onset of urinary elimination [91]. The metabolite with the best pharmacokinetics is RSV-3-sulfate. It has a peak plasma level of 14 µM after ingestion of five grams of RSV, with an area under the curve (AUC) over 24 h of follow-up that is approximately 20-fold higher than RSV due to the rapid urinary excretion of RSV and its metabolites [91]. RSV also undergoes enterohepatic circulation since a second plasma peak can be observed 5 h after ingestion, and it is found in the feces [91,92]. These data thus highlight that tissues are exposed to small quantities of RSV for a limited time, which is not the case for its metabolites [77]. As for toxicity, the threshold is not known exactly, but a trial with rats showed no major hepatic or renal problems up to 300 mg/kg/day [105]. In humans, no major effects were observed for single doses up to five grams [106,107,108,109].

Pharmacokinetic studies suggest that peak plasma levels, in the order of 9 µM free RSV and 680 µM total RSV, can be achieved in animals after administration of high but pharmacologically acceptable doses (100 mg/kg/day of pure RSV) [77]. Regarding the tissue accumulation and bioavailability of RSV and its metabolites, it seems that they are preferentially found in organs and fluids related to absorption and elimination, such as the stomach, intestine, liver, kidney, bile, and urine [98,110,111]. Nevertheless, despite low plasma concentrations, RSV administration has undeniable biological effects on many in vivo models [112,113,114,115].

### 5.2. Epidemiological and Interventional Studies in Humans

Some studies have been carried out in humans in order to identify the pharmacokinetics and biological effects of RSV [78,79,91,92,95,99,100,101,102]. Preclinical in vivo studies (Table 3) indicate that RSV could be a promising molecule in the prevention and treatment of certain cancers. As an extension of these in vivo data, a 10-year epidemiological study of 369 cases and 602 controls showed a reduction of at least 50% in the risk of breast cancer in women consuming RSV from grapes (not wine) [48]. Nearly 10 phase I, II, and III clinical trials of oral consumption of RSV as a pure compound or RSV-rich products (grapes and grape juice) are currently underway (Table 3). Among these clinical trials, two studies are investigating the effects on the metabolism of healthy subjects (phase I and II enzymes), the identification of metabolites, and the safety of the molecule (Identifier: NCT00721877 and 00098969). Other studies (one phase I, the other phase III) that are interested in anti-neurodegenerative properties are studying the impact of RSV or a drug containing RSV on patients suffering from moderate Alzheimer’s disease (Identifier: NCT00743743 and 0008678431). A phase II study initiated by the University of California is assessing the effect of RSV on metabolic regulation in patients with metabolic syndrome, and another phase I study is looking at the beneficial effects of RSV in caloric restriction in post-menopausal women (Identifier: NCT00654667 and 00823381) The other four clinical studies are more specifically focused on RSV for chemopreventive or therapeutic use. One phase II study is being done in follicular lymphoma (Identifier: NCT0455416). In this study, various groups are formed according to natural compounds (e.g., catechin, RSV, alicin) with the aim of measuring the rate of proliferation and the production of pro-inflammatory cytokines. The other studies will try to show if RSV can modulate the expression of COX-2 (Identifier: NCT00433576) and the Wnt pathway (Identifier: NCT00256334) in patients with colorectal cancer. The same type of study on the modulation of the Wnt/-catenin pathway (Identifier: NCT00578396) will be performed on healthy subjects.

Thus, despite its low bioavailability, RSV has demonstrated a clear effect in many animal models. Some of these effects have also been found in humans, but it is necessary to wait for the publication of ongoing and future clinical trials in order to have a clearer understanding of its therapeutic efficacy in combination with chemotherapy.

## 6. Conclusions

The prescription of immunotherapies is growing exponentially. Like all targeted therapies, immunotherapies are expensive. The average price per treatment varies, depending on the antibody, from EUR4000 to more than EUR12,000. However, it is impossible to assess the overall cost of the treatment since it is administered intravenously, every 2 to 3 weeks “until disease progression or the appearance of unacceptable toxicity”. Nevertheless, immunotherapies have proven their superiority over conventional chemotherapy or targeted therapy treatments. A recent study has shown that they can keep the disease under control for a longer period of time in twice as many patients compared to conventional treatments with chemotherapy or targeted therapies. Unfortunately, the proportion of “responder” patients—that is to say, in whom the treatment is effective—varies considerably from one cancer to another. It can reach 40% in melanoma and is between 20% and 30% in the lung, but only 1% of patients with pancreatic cancer are responders [116,117]. This disparity could be explained by the fact that the immune system must recognize the tumor as a foreign body that needs to be eliminated. So the more mutated the tumor is, the more the immune system will attack it. Immunotherapy is, therefore, more effective in lung cancer and melanoma because they are mostly caused by mutagens: cigarette smoke and ultraviolet rays. In addition, while immunotherapy is not effective in some patients, it can be fatal in others. This is the case with so-called “hyper-progressive” patients [116,117]. In these patients, the antibodies cause an acceleration of tumor growth that can lead to death. Depending on the type of cancer, this phenomenon is observed in 9% to 29% of cases. In non-small cell lung cancer, hyper-progression has been shown in 1 in 7 patients, which is twice as often as with chemotherapy. Researchers have also observed cases of pseudo-progression: the tumor begins to grow—under the influence of the infiltration of cells of the immune system—before regressing [118,119]. To date, oncologists are unable to predict whether the increase in tumor size is indicative of hyper-progression or pseudo-progression. Faced with the low rate of “responders” and the high cost of injections, the main challenge for immunotherapies will be to select the patients in whom the treatment is likely to be effective or to develop new strategies to enhance the efficiency of immunotherapy, perhaps to block the hyper-progression. Certain molecules with pleiotropic effects, such as RSV, have been proposed for this purpose. Following publications relative to the beneficial effects of RSV, it has become a popular dietary supplement. However, the data in the literature highlighted herein calls for caution: depending on the cell type, the type of cancer, and the dose consumed, the effects of RSV are uncertain. Additional studies must be carried out to determine when this polyphenol increases the expression of PD-L1 on the surface of cells and whether PD-L1 is still functional or not. Another very interesting research perspective would be to determine, as is the case with chemotherapy, whether co-administration of nivolumab or atezolizumab with RSV can increase the efficacy of immunotherapy and counteract the phenomenon of hyper-progression.

## Figures and Tables

**Table 1:** Dosages of RSV and its metabolites in plasma after oral administration.

Species	Dose	Plasma Peak		References
		Free RSV	Conjugated-RSV	
Rat	50 mg/kg	6.6 µM	105 µM (glucuronide)	[93]
	20 mg/kg	1.2 µM		[94]
	2 mg/kg	0.09 µM	1.2 µM (total)	[95]
	5 mg/kg	0.11 µM	1.5 µM (total)	
	2 mg/kg	0.77 µM		[96]
Mice	20 mg/kg	2.6 µM		[94]
	20 mg/kg	Traces	~5 µM (sulfate)	[97]
			~1 µM (glucuronide)	
	60 mg/kg	Traces	~300 µM (sulfate)	
			~170 µM (glucuronide)	
	240 mg/kg	32 µM		[98]
Rabbit	20 mg/kg	1.1 µM		[94]
Human	25 mg/70 kg	~37 nM	~2.1 µM (total)	[78]
	25 mg/personne	<22 nM	~2.1 µM (total)	[92]
	0.5 g/person	0.3 µM	3.7 µM (sulfate)	[91]
			1.9 µM (glucuronides)	
	1 g/person	0.5 µM	7.1 µM (sulfate)	
			2.9 µM (glucuronides)	
	2.5 g/person	1.1 µM	9.1 µM (sulfate)	
			6.2 µM (glucuronides)	
	5 g/person	2.4 µM	14 µM (sulfate)	
			7.5 µM (glucuronides)	

**Table 2:** Clinical studies of RSV bioavailability.

Human Subjects	Objectives	Dose	Administration	Result	References
Healthy humans (10)	Bioavailability	25 mg/person	Oral	Plasmatic peak of free and conjugated forms: 30 min ~25% found in urine in 24 h	[79]
Healthy humans (12)	Bioavailability	25 mg/70 kg	Oral	Peak of conjugated glucuronides and sulfates in serum at 30 min; urinary excretion at 24 h: 16–17% of the dose	[78]
Healthy men (3)/Healthy women (3)	Bioavailability	25 mg/person	Oral, i.v.	Absorption: 70%; t1/2 plasmatic: 9.2 h; mainly excreted in urine	[92]
Healthy humans (3)	Bioavailability	0.03; 0.5; 1 mg/kg	Oral	RSV free and derived detectable in plasma and urine; 25–50% of RSV found in urine during 24 h	[95]
Healthy humans (4)	Bioavailability	1 g/person	Oral	Six conjugated metabolites are detected in serum and urine	[99]
Healthy humans (40)	Phase I dose and pharmacokinetic studies	0.5; 1; 2.5; 5 g/person	Oral	No toxicity observed; plasmatic peak at 1.5 h; 77% of all forms are excreted in urin in 24 h	[91]
Healthy men (14)/Healthy women (11)	Bioavailability	3.4; 7.5; 33 µg/kg	Oral	RSV aglycone and its glucuronides are found in serum: The meal dose does not affect the bioavailability	[100]
Healthy men (14)/Healthy women (11)	Urine excretion	0.36; 0.4; 2.6 mg/person	Oral	Increase in total metabolites	[102,104]

( ) The number of subjects is indicated in parentheses.

**Table 3:** Clinical trials in humans.

Clinical Trial Identification	Phase	Sponsor	Official Title	Dose	Objectives	Inclusion Criteria
NCT00721877	I	University of Arizona; NCI (National Cancer Institute)	“Clinical study of resveratrol in drug and carcinogen metabolizing enzymes”	Oral, Once/J/4 weeks	1. To determine the effects of RSV on the p450 activity of healthy adults 2. Determine the effects on phase II enzymes 3. Evaluate the safety of the molecule	Healthy individuals
NCT00256334	II	University of California; Irvine	“Resveratrol for patients with colon cancer”	1st group: 20 mg/day RSV pill; 2nd group: A dose of 80 mg/day; 3rd group: A dose of 160 mg/day	To test whether RSV modulates the Wnt pathway in colon cancer and normal colonic mucous membranes	1. Patients diagnosed with colon cancer 2. Patients with a surgical resection plan
NCT00654667	III	University of California, San Francisco	“Mechanism of metabolic regulation of resveratrol on humans with metabolic syndrome”	1st group: 5 g RSV/D/1 month; 2nd group: 5 placebo capsules/D/1 month	1. Measurement of insulin sensitivity 2. Measurement of cholesterol metabolism, fat mass, BMI, quality of life 3. Measurement of physical activity 4. Assess appetite and satiety 5. Measurement of biomarkers (leptin, ALAT, ASAT, etc.)	1. Adult aged 50 and over 2. Women aged 50 and over, post-menopausal 3. BMI 25 to 35 4. HOMA-IR plasma glucose score and serum insulin levels >2.7 5. Food >40% of calories from fat 6. Sedentary
NCT00743743	III	Medical College of Wisconsin	“Pilot study of the effects of resveratrol supplement in mild-to-moderate Alzheimer’s disease”	1st group: 1 capsule (Longevinex®) of 215 mg/day/52 weeks; 2nd group: 1 placebo capsule/D/52 weeks	To determine the effects of supplementation with wine extract (in OTC) on the cognitive and overall functions of patients with moderate or moderate Alzheimer’s disease.	1. Post-menopausal men or women with a probable clinical diagnosis of Alzheimer’s 2. Subjects with moderate Alzheimer’s disease 3. Ischemia Hachinski
NCT00098969	I	University of Michigan (NCI)	“Phase I single-dose safety and pharmacokinetics clinical study of resveratrol”	Cohort of 10 subjects receiving doses of RSV up to the maximum tolerated dose (MTD). The subjects receive the oral dose of RSV once in 1 day and are then followed for 7 days.	1. Determine the concentration of RSV and its metabolites in the plasma, urine, and feces of healthy subjects 2. Correlate the dose with the systemic concentration of the drug and its metabolites in each participant. 3. Evaluate the safety of the drug	Healthy subjects
NCT00433576	I	University of Michigan (NCI)	“Phase I repeat-dose study of resveratrol in colorectal cancer patients: tolerability, target tissue levels and pharmacodynamics”	Level 2 patients receive oral RSV over 8 days and undergo a colorectomy on day 9	1. To determine the relationship between the oral dose of RSV and the levels of RSV and its metabolites in colonic mucous in patients with “resectable” colorectal cancer. 2. To determine the relationship between the levels of RSV and colonic mucosal metabolites and plasma concentrations in patients. 3. Measurement of COX-2 expression in colorectal cancerous tissue before and after treatment 4. Measurement of the concentration of mG1 in cancerous colorectal tissue 5. Determination of the toxicity profile of the molecule	Level 1: colorectal endoscopy; patients whose biopsy confirms continuous colorectal adenocarcinoma at level 2 Level 2: colorectal adenocarcinoma, planning for colorectomy
NCT00578396	I	University of California; Irvine	“Phase I biomarker study of dietary grape-derived low dose resveratrol for colon cancer prevention”	Low doses of RSV	Measurement of the expression and cellular localization of β-catenin in the intestinal mucosa and of the expression of target genes of the Wnt pathway Determine if grape food supplementation affects the proliferation of cells in the intestinal mucosa	Healthy subjects
NCT008678431	III	Department of Veterans “Alzheimer Association Mount Sinai School of Medicine”	“A single-center, multi-site, randomized, double-blind, placebo-controlled trial of resveratrol with glucose and malate (RMG) to slow the progression of Alzheimer’s disease”	1st group: Food supplement in the form of grape juice: RSV with glucose and malate 2nd group: placebo		50 to 90 years Alzheimer’s disease
NCT00823381	I	University of California; Irvine	“Effect of Resvida™”	1st group: one Resvida pill (75 mg of RSV) once 1 day with breakfast 2nd group: A placebo pill with breakfast 3rd group: calorie restriction: 30% reduction in calorie intake	- Compare the beneficial effects of both RSV and calorie restriction - Determine if RSV mimics any effects of calorie restriction.	Subjects 35 to 70 years post-menopausal women
NCT00455416	II	University of Oslo; Rikshospitalet HF	“Dietary intervention in stage III/IV follicular lymphoma. Impact on markers of cell proliferation, apoptosis, host immune cell infiltrate and oxidative stress”	1st group: omega 3 (EPA et DHA) 2nd group: Selenium 3rd group: Garlic extract (Allicin) 4th group: Apple juice (ellagic acid) 5th group: grape juice (RSV, quercetin) 6th group: green tea (epigallocatechin)	Measurement of apoptosis and the rate of proliferation of tumor cells. Measurement of pro-inflammatory cytokine levels	18 years or older Histologically verified grade I or II follicular lymphoma, without clinical sign of transformation into aggressive lymphoma Stage II/IV
NCT02261844	I/II	University of Louisville	“Resveratrol and Human Hepatocyte Function in Cancer	Dietary supplement: RSV RSV 1 gm po × 10 days prior to liver resection Other name: Biotivia Drug: placebo Placebo 1 pill daily × 10 days	Determine if RSV, a nutritional supplement, shows a beneficial effect in the cellular function of normal liver cells and diseased liver cells (cancer cells) in samples of liver tissue taken during elective liver surgery.	Undergoing elective liver resection for liver cancer
NCT01476592		University of Wisconsin	“A Biological Study of Resveratrol’s Effects on Notch-1 Signaling in Subjects With Low-Grade Gastrointestinal Tumors”	5 gm/day of RSV orally, in two divided doses of 2.5 gm each without a break in therapy for a total of three cycles.	Examine the effects of RSV and Notch-1 on neuroendocrine tumor tissue and to examine how people with neuroendocrine tumors who take RSV for up to three months tolerate the product.	Age >18 years old Women who are not post-menopausal must have a negative enrollment blood test and agree to use an effective mode of contraception while taking the study medication. Greater than four weeks must have elapsed since any previous therapy was administered for the neuroendocrine tumor, including surgery, radiation, chemotherapy, or local liver therapy. Octreotide use is allowed but must be initiated at least four weeks prior to enrollment and the pre-treatment biopsy. Able to give informed consent and willing to undergo the post-treatment research biopsy. Must be able to take oral medications and be without GI tract obstructive symptoms.
NCT00920803	I	GSK Investigational Site; Leicester, Leicestershire, United Kingdom	“A Phase 1, Double-Blind, Randomized Clinical Study to Assess the Safety, Pharmacokinetics, and Pharmacodynamics of SRT501 in Subjects With Colorectal Cancer and Hepatic Metastases”	5.0 g of SRT501 (a RSV formulation) will be administered once daily as an oral reconstituted powder for 14 days at the same time each day. On Days 1 and 2, SRT501 will be administered for approximately 15–30 min following the consumption of a standardized breakfast. On all other days, SRT501 will be administered for approximately 15–30 min following the consumption of the evening meal. Following the course of SRT501 administration, subjects will undergo scheduled surgical removal of their metastatic liver disease as well as non-diseased tissue. Due to scheduling and surgical availability, subjects can receive SRT501 for a minimum of 10 days and a maximum of 21 days.	Assess the safety, pharmacokinetics, and pharmacodynamics of SRT501 in subjects diagnosed with colorectal cancer and hepatic metastases.	Be male or female, older than 18 years of age. Have histologically or cytologically confirmed and diagnosed colorectal cancer with hepatic metastases. Have not received chemotherapy or anti-neoplastic therapy for a malignancy within six weeks of first dose of SRT501 or placebo. Have a life expectancy of greater than 3 months.
